# Vanillin Affects Amyloid Aggregation and Non-Enzymatic Glycation in Human Insulin

**DOI:** 10.1038/s41598-017-15503-5

**Published:** 2017-11-08

**Authors:** Clara Iannuzzi, Margherita Borriello, Gaetano Irace, Marcella Cammarota, Antimo Di Maro, Ivana Sirangelo

**Affiliations:** 1 Department of Biochemistry, Biophysics and General Pathology, Università degli Studi della Campania “Luigi Vanvitelli”, Naples, 80138 Italy; 2 Department of Experimental Medicine, Università degli Studi della Campania “Luigi Vanvitelli”, Naples, 80138 Italy; 3 Department of Environmental, Biological and Pharmaceutical Sciences and Technologies, Università degli Studi della Campania “Luigi Vanvitelli”, Caserta, 81100 Italy

## Abstract

Curcumin is known for its anti-inflammatory, antioxidant and anticancer activity, as well as for its ability to interfere with amyloid aggregation and non-enzymatic glycation reaction, that makes it an attractive potential drug. However, curcumin therapeutic use is limited because of its low systemic bioavailability and chemical stability as it undergoes rapid hydrolysis in physiological conditions. Recently, much attention has been paid to the biological properties of curcumin degradation products as potential bioactive molecules. Between them, vanillin, a natural vanilla extract, is a stable degradation product of curcumin that could be responsible for mediating its beneficial effects. We have analyzed the effect of vanillin, in comparison with curcumin, in the amyloid aggregation process of insulin as well as its ability to prevent the formation of the advanced glycation end products (AGEs). Employing biophysical, biochemical and cell based assays, we show that vanillin and curcumin similarly affect insulin amyloid aggregation promoting the formation of harmless fibrils. Moreover, vanillin restrains AGE formation and protects from AGE-induced cytotoxicity. Our novel findings not only suggest that the main health benefits observed for curcumin can be ascribed to its degradation product vanillin, but also open new avenues for developing therapeutic applications of curcumin degradation products.

## Introduction

Insulin, a key hormone regulating glucose homeostasis, is stored in the pancreas as inactive zinc hexamer; when released into the blood serum, the hexameric form dissociates into dimers and subsequently, into monomers, which are the physiologically active forms^1^. Monomeric and dimeric forms of insulin are less stable than hexamer and tend to aggregate forming amyloid fibrils^2–5^. Amyloid aggregation is associated with several pathological conditions including neurodegenerative diseases, such as Alzheimer and Parkinson, infectious prion disease, non neuropathic systemic amyloidosis and type 2 diabetes^6^. Amyloid fibrils are characterized by the cross-β-structure, a common structural motif in which individual strands in the β-sheets run perpendicular to the long axis of the fibrils. Amyloid fibrils are formed by a stepwise process via oligomerization, nucleation and growth phase. The nucleation is the slower step, while the growth phase proceeds quickly as soon as the nuclei are formed^7^. *In vitro* experiments have revealed that also proteins with no link to pathological conditions can form amyloid structures, supporting the idea that amyloid formation may be a generic property of all polypeptides^6^. The propensity to form amyloid fibrils depends on the protein sequence and environmental conditions such as temperature, solution milieu, pH, and interaction with lipid interfaces^6^. So far, no pathogenic fibrillar assembly has been found *in vivo* for human insulin. However, pathological conditions related to insulin fibril formation can occur in patients affected by type 2 diabetes. In fact, insulin is able to form amyloid-like fibrils in the site of medication injections of insulin-dependent diabetic patients causing a pathological condition called insulin injection amyloidosis^8–12^. In this pathology, insulin amyloid fibrils form a hard subcutaneous mass at the injection site and an immune response may be triggered. Also, the insulin fibril formation can cause serious therapeutic problems such as poor glycemic control because of the impairment in insulin absorption, and catheter occlusions during continuous subcutaneous insulin infusion^13,14^.

Insulin is susceptible, especially in diabetic conditions, to non-enzymatic glycation. Protein glycation involves the reaction between reducing sugars and free amino groups in amino acid side-chains^15–17^. Advanced glycation end products (AGEs) are the end products of the glycation reaction and their accumulation has been suggested to be the main factor responsible for the development and the progression of several diabetic complications including nephropathy, retinopathy and neuropathy^18–25^. In addition, AGEs have been linked to amyloid-based neurodegenerative disease^26–28^. Glycation of insulin is known to differentially affect its amyloid aggregation process depending on the glycating agent^29–31^. We have recently reported that insulin glycation by D-ribose, although preventing amyloid aggregation, strongly affects the cell viability through the AGEs formation^31^.

Significant efforts have been addressed to the study of anti-amyloidogenic and anti-AGE agents with the aim of developing new potential therapeutic strategies in amyloid-based neurodegenerative disease^32,33^. In this respect, curcumin, a natural phenol abundant in turmeric, has been shown to inhibit amyloid aggregation in several proteins like Aβ-peptide, human islet amyloid polypeptide, α-synuclein, hen egg-white lysozyme and bovine insulin^34–42^. Curcumin is also known for many other properties including anti-inflammatory, antioxidant, anticancer and anti-AGE activity, that make it an attractive potential drug^43–46^. Nevertheless, its potential therapeutic use seems to be limited because of its very low systemic bioavailability after oral administration owing to its low water solubility and its chemical instability under physiological or alkaline conditions^47,48^. Indeed, curcumin undergoes rapid hydrolysis followed by molecular fragmentation at physiological pH and clinical trials have found undetectable *in vivo* levels of curcumin after administration of high doses of this compound^49,50^. To overcome this issue, different delivery systems such as micelles, liposomes, nanoparticles, or synthesis of more stable curcumin derivatives have been tested^51–53^. Despite the high instability, curcumin is known to lead to clear health benefits. Recently, much attention has been paid to the biological properties of the degradation products of the curcumin^51,54–56^. Analyzing the similarities between the biological activity of curcumin and its degradation products against cancer and Alzheimer’s disease, it has been suggested that the bioactive degradation products may contribute to the pharmacological effects of curcumin^55,57^. Due to the high degradation rate of curcumin, its degradation products are expected to have high concentration in the blood thus augmenting their pharmacological effects. Thus, the bioactive degradation products might act as important mediators for the pharmacological effects of curcumin^55^. Vanillin is one of the main degradation products of curcumin^49,55,58^. An *in vivo* study has revealed that, upon curcumin consumption, ferulic acid and vanillic acid (possibly the enzymatic oxidation product of vanillin in liver) are the major metabolites and their concentrations are up to 1000-fold higher than those of curcumin, especially in urine^55,59^. Vanillin is the major component of vanilla bean extract commonly used as a natural flavoring agent and known for its anti-proliferative and antioxidant activity^60,61^. Recently, it has been reported that vanillin is able to restrain non-enzymatic glycation and AGE-related amyloid aggregation in albumin^62^.

In the light of these considerations, in the present study we have investigated the effect of vanillin, in comparison with curcumin, in the amyloid aggregation process of human insulin as well as its anti-AGE activity. The results indicate that vanillin and curcumin similarly affect insulin amyloid aggregation. Moreover, vanillin showed anti-AGE activity as well as a protective effect on the AGE toxicity as already reported for curcumin. These results support the hypothesis that the main health benefits observed for curcumin can be mediated by its degradation product vanillin.

## Results

### Curcumin effect on human insulin amyloid aggregation and amyloid toxicity

Insulin has been widely used as model protein in the study of amyloid formation as, under specific experimental conditions, it is highly prone to form amyloid fibrils^63–65^. The rate of insulin amyloid fibril formation is affected by several factors, such as pH, temperature, protein concentration, ionic strength, and presence of denaturants^66,67^. Taking into account the importance of physiological pH and temperature, in this study the aggregation of insulin was performed incubating the protein at pH 7.0 under stirring with teflon ball at 37 °C.

**Figure 1:**
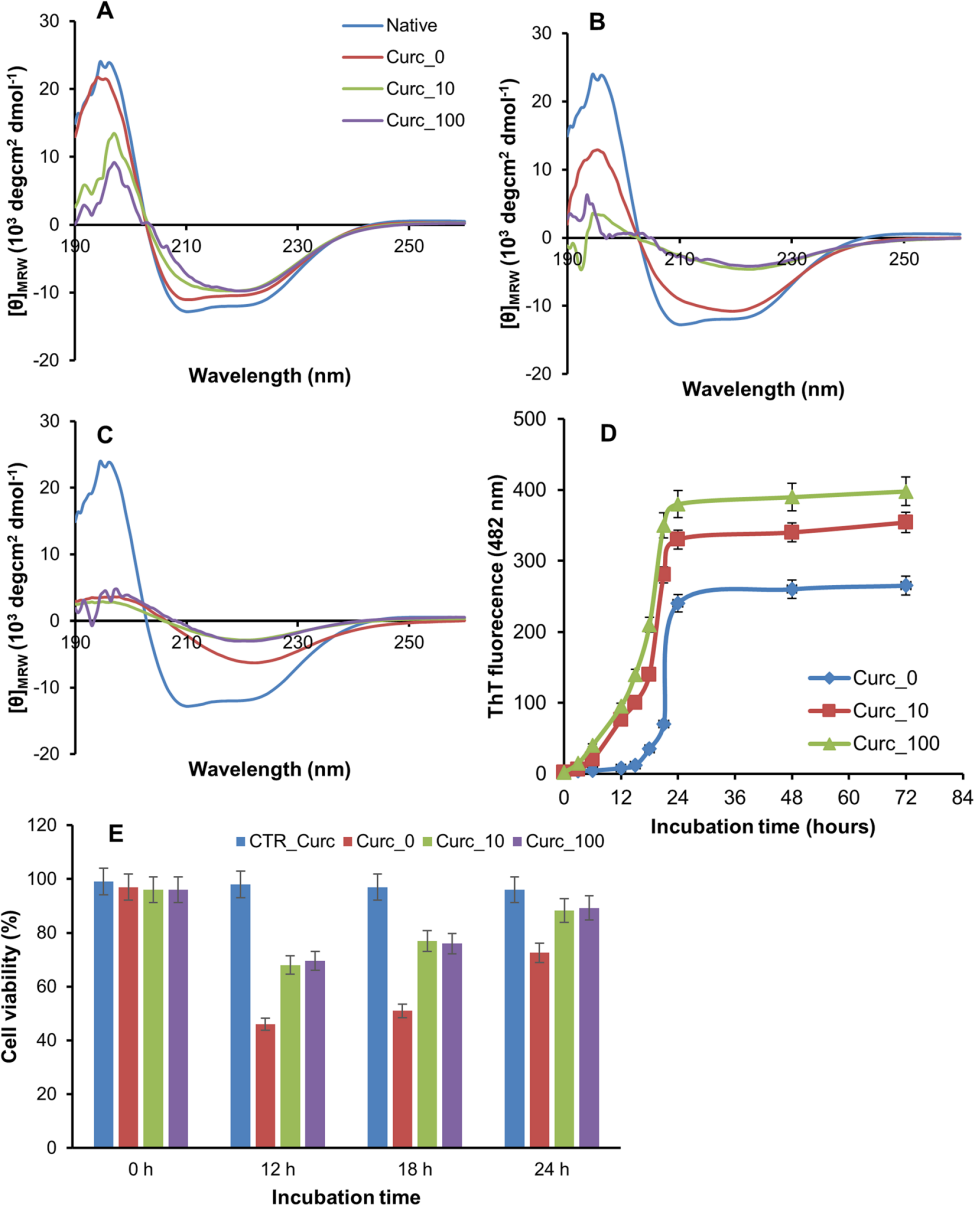
Effect of curcumin on insulin amyloid formation and cytotoxicity. The effect of curcumin on insulin amyloid formation was assayed by far-UV CD spectroscopy and ThT fluorescence at different times of incubation in aggregating conditions. In panels A–C are reported the CD spectra of insulin in the absence and in the presence of curcumin (10 and 100 µM) at 12 (**A**), 18 (**B**) and 24 hours (**C**) of incubation in comparison to the native protein. The ThT fluorescence emission was recorded at 482 nm upon excitation at 450 nm (**D**). Protein concentration were 0.3 mg/mL and 8 µM for CD and ThT measurements, respectively. (**E**) Cytotoxicity of insulin amyloid aggregates formed in the presence of curcumin. SH-SY5Ycells were exposed for 24 hours to insulin incubated for 0, 12, 18 and 24 hours in aggregating condition in the absence and in the presence of curcumin (10 and 100 µM) and cell viability was evaluated by MTT assay. Data are expressed as average percentage of MTT reduction ± SD relative to control cells from triplicate wells from 5 separate experiments (p < 0.01). CTR_Cur represents cell exposed to curcumin at the higher working concentration. Other experimental details are described in the Methods section.

To evaluate the effect of curcumin on insulin amyloid formation, we performed far-UV CD spectroscopy and Thioflavin T (ThT) fluorescence analysis at different times of incubation in aggregating conditions (Fig. 1). Curcumin concentrations were chosen in the 10^−6^ M range as this was suggested to be comparable with physiological concentration achieved by curcumin in the central nervous system by oral dosing^68^. Insulin amyloid formation occurs through an elongation process, the early prefibrillar aggregates (oligomers and protofibrils) are mainly characterized by an α-helical structure, while only amyloid fibrils show the typical cross-β-structure^69,70^. In order to monitor structural transitions, far-UV CD spectra of insulin were recorded in the absence and in the presence of curcumin at 12, 18 and 24 hours of incubation in aggregating conditions (Fig. 1A–C). As expected, the CD spectra recorded at the beginning of the aggregation process (time 0) showed no differences between the samples incubated in the absence and in the presence of curcumin (data not shown). After 12 hours of incubation, the spectrum of insulin in aggregating conditions almost resembled that of the native protein showing two minima at 222 nm and 208 nm, and a positive signal around 195 nm consistent with the presence of α-helical conformation likely associated to the early aggregating species. At 18 hours of incubation the spectrum displayed a decrease in the ellipticity at 208 nm suggesting that an α to β-transition was taking place. At 24 hours of incubation, the CD spectrum was characterized by a clear minimum at around 218 nm characteristic of extensive β-sheet structures. These data indicate that, after 24 hours of incubation in aggregating conditions, insulin undergoes a conformational transition from α-helix to β-sheet structure associated to the amyloid fibril formation. Differently, the α to β-transition was already observed at 18-hour incubation for samples incubated in the presence of curcumin indicating that it affects insulin aggregation kinetics accelerating the amyloid fibrils formation.

The effect of curcumin on insulin amyloid fibril formation was also evaluated by ThT binding assay. ThT is an amyloid fibril-specific dye as, when bound to the cross-β amyloid structure, it shows a strong fluorescence intensity^71^. In Fig. 1D is reported the insulin aggregation kinetics in the presence and in the absence of curcumin monitored by ThT fluorescence. Samples incubated in the presence of curcumin showed a shorter lag phase and a higher ThT intensity at the end of the process at the tested concentrations thus indicating that curcumin accelerates insulin amyloid aggregation and favors fibril formation in aggregating conditions. Moreover, as additional control we monitored the protein concentration in the soluble fraction during the aggregation process by absorption spectroscopy and SDS-PAGE analysis (Supplementary Material Fig. S1). The results indicate that the amount of soluble protein decreases faster in the presence of curcumin. There are some concerns about the employment of ThT to detect fibril formation in the presence of curcumin, they are related to the superimposition of curcumin and ThT fluorescence emission spectra and to the ability of curcumin to compete with ThT for fibril binding^72^. This aspect should be considered when inhibitory effects are observed as they can yield false positive. However, in our case, ThT assay provides reliable information as we observe an increase in the intensity of fluorescence in the presence of curcumin. Moreover, no fluorescence emission was detected in samples incubated in the presence of curcumin before the addition of ThT.

Amyloid aggregates are known to affect cell toxicity; it is well established that the amyloid cytotoxicity is associated to the early soluble oligomeric aggregates whereas amyloid fibrils are essentially harmless^73^. To assess the cytotoxicity of insulin aggregates formed in the presence of curcumin, we evaluated the cell viability by the MTT assay in SH-SY5Y neuronal cells (Fig. 1E). This cellular model was chosen as amyloid toxicity is generally associated to neurodegeneration and, also, this cell line is widely used for the evaluation of cytotoxicity in amyloid aggregates. To this aim, cultured human neuroblastoma SH-SY5Ycells were exposed to insulin incubated for 12, 18 and 24 hours in aggregating condition in the absence and in the presence of curcumin. As control, the cytotoxicity of insulin at the beginning of the aggregation process (time 0) was tested and no toxicity was observed both in the absence and in the presence of curcumin. As expected, in the absence of curcumin, early amyloid aggregates (12 hours) reduced cell viability by approximately 65% compared to untreated cells while amyloid fibrils (24 hours) were almost harmless^31^. Interestingly, samples aggregated in the presence of curcumin for 12 hours showed a reduced toxicity likely associated to a greater amount of harmless amyloid fibrils. These data suggest that curcumin, accelerating the amyloid fibrils formation, could have a protective effect on insulin amyloid toxicity.

Differently from data obtained for other amyloid proteins, in which curcumin showed anti-amyloid activity^34–42^, our data indicate that curcumin accelerates amyloid aggregation in human insulin at physiological pH thus favoring the formation of harmless fibrils. The effect of curcumin on the amyloid aggregation process could depend on the conformational organization of the model protein. Moreover, considering the high instability of curcumin under neutral pH, it is likely that the effect that we have observed is not caused by curcumin itself but mainly due to its degradation products^49,74^. For this reason, we performed the same experiments in the presence of vanillin, one of the main degradation products of curcumin at neutral pH.

### Vanillin effect on human insulin amyloid aggregation

**Figure 2:**
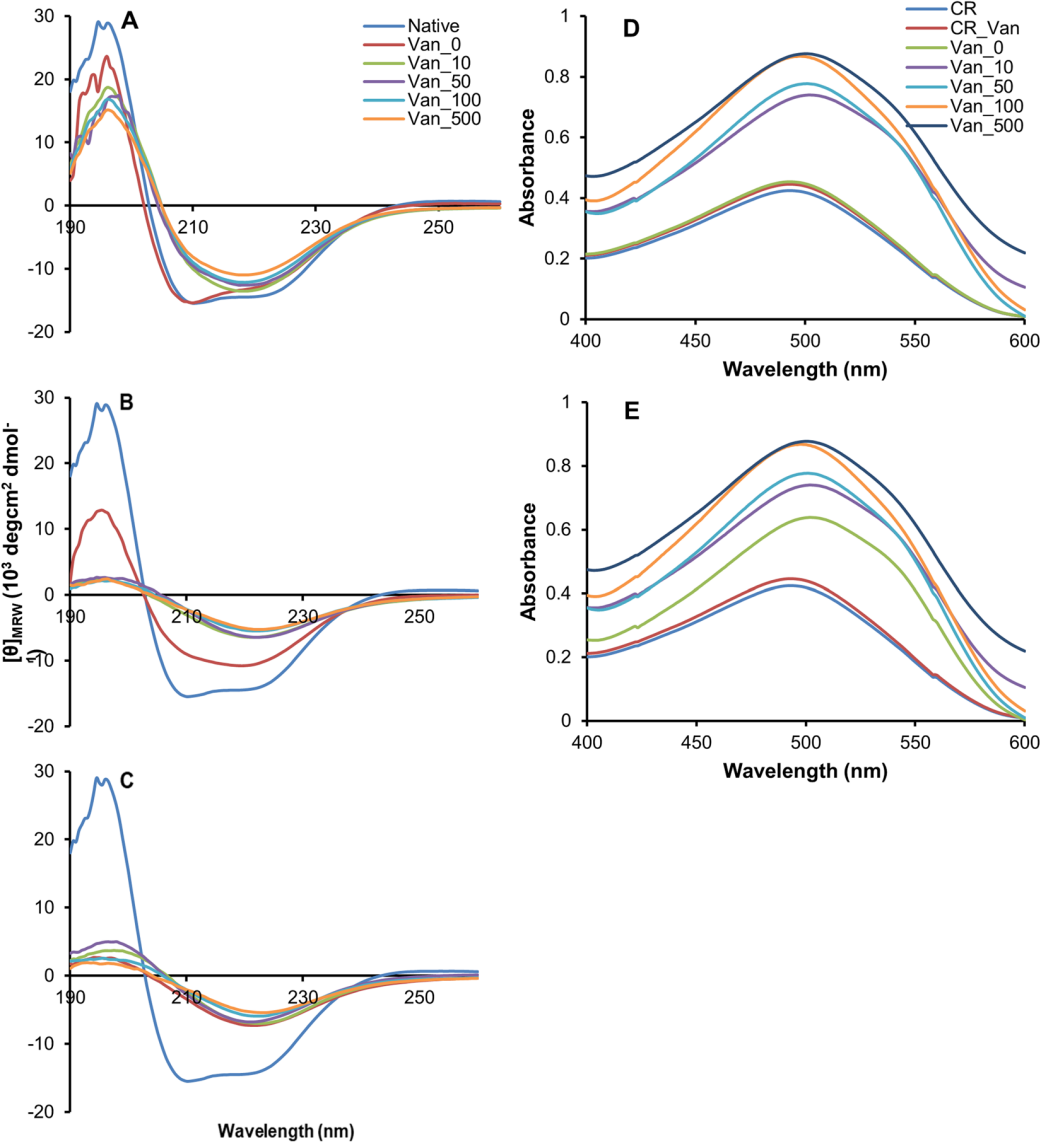
Effect of vanillin on insulin amyloid formation. CD spectra of insulin in aggregating conditions in the absence and in the presence of vanillin (10–500 µM) at 12 (**A**), 18 (**B**) and 24 hours (**C**) of incubation in comparison to the native protein. Protein concentration was 0.3 mg/mL. Absorption spectra of 5 µM CR bound to 8 µM insulin samples aggregated in the presence and in the absence of vanillin for 18 (**D**) and 24 hours (**E**) in comparison to the free CR. CR_Van represent the spectra of CR in the presence of vanillin at the higher working concentration. Other experimental details are described in the Methods section.

To evaluate the effect of vanillin in insulin amyloid formation, we tested the ability of insulin to form amyloid aggregates in physiological conditions in the presence of vanillin. To this aim, human insulin was incubated in the presence and in the absence of different concentrations of vanillin (10, 50,100, and 500 µM) and samples were analyzed by far-UV CD spectroscopy at 12, 18 and 24 hours of incubation in aggregating conditions (Fig. 2A–C). The CD spectra recorded at the beginning of the aggregation process (time 0) indicated no variations between the samples incubated in the absence and in the presence of vanillin. After 12 hours of incubation, while the sample incubated in the absence of vanillin was still mainly in a α-helical conformation, samples incubated in the presence of vanillin displayed a decrease in the ellipticity at 208 nm suggesting that a α to β-transition was taking place. At 18 hours of incubation, while the spectrum of insulin alone indicated that an α to β-transition was taking place, the spectra recorded in the presence of vanillin at all concentrations tested showed a clear shift to around 218 nm suggesting that the α to β-transition was completed. At 24 hours, all spectra were overlapping thus indicating that an extensive β-sheet structure was formed also for insulin in the absence of vanillin.

When the effect of vanillin in insulin amyloid fibril formation was evaluated by ThT assay, the presence of vanillin was affecting the ThT emission, i.e. samples in the presence of vanillin were showing fluorescence at 482 nm (upon excitation at 350 nm) even before the addition of ThT. For this reason, the amyloid fibril formation was evaluated by Congo Red (CR) binding assay, a widely used method to detect amyloid fibrils. Indeed, while the free CR shows a maximum at 490 nm in the absorption spectrum, when it is bound to *β*-sheet-rich amyloid fibrils, a characteristic increase in absorbance accompanied by a red shift in the absorption maximum from 490 to 540 nm occurs^75^. Figure 2D–E shows the absorption spectra of CR bound to insulin samples aggregated in the absence and in the presence of vanillin for18 and 24 hours in comparison to that of free CR. The absorption spectra were first recorded at the beginning of the aggregation process (time 0) and no variations were observed both in the absence and in the presence of vanillin. As control, we recorded the CR spectrum in the presence of vanillin at the higher working concentration which was indistinguishable from that of CR alone. At 18 hours incubation time, the CR spectrum recorded for insulin sample in the absence of vanillin was almost superimposed to that of free CR, thus indicating that amyloid fibrils were not yet formed (Fig. 2D). Differently, the spectra recorded for insulin in the presence of vanillin showed a red shift at any concentration and a concentration-dependent increase in absorbance indicative of the presence of amyloid structures. At 24 hours, also the spectrum of insulin incubated in the absence of vanillin showed the typical red shift and absorption increase as expected. These results are in perfect agreement with the CD data and suggest that vanillin, as well as curcumin, strongly affects insulin aggregation kinetics accelerating the amyloid fibrils formation (Fig. 2E).

**Figure 3:**
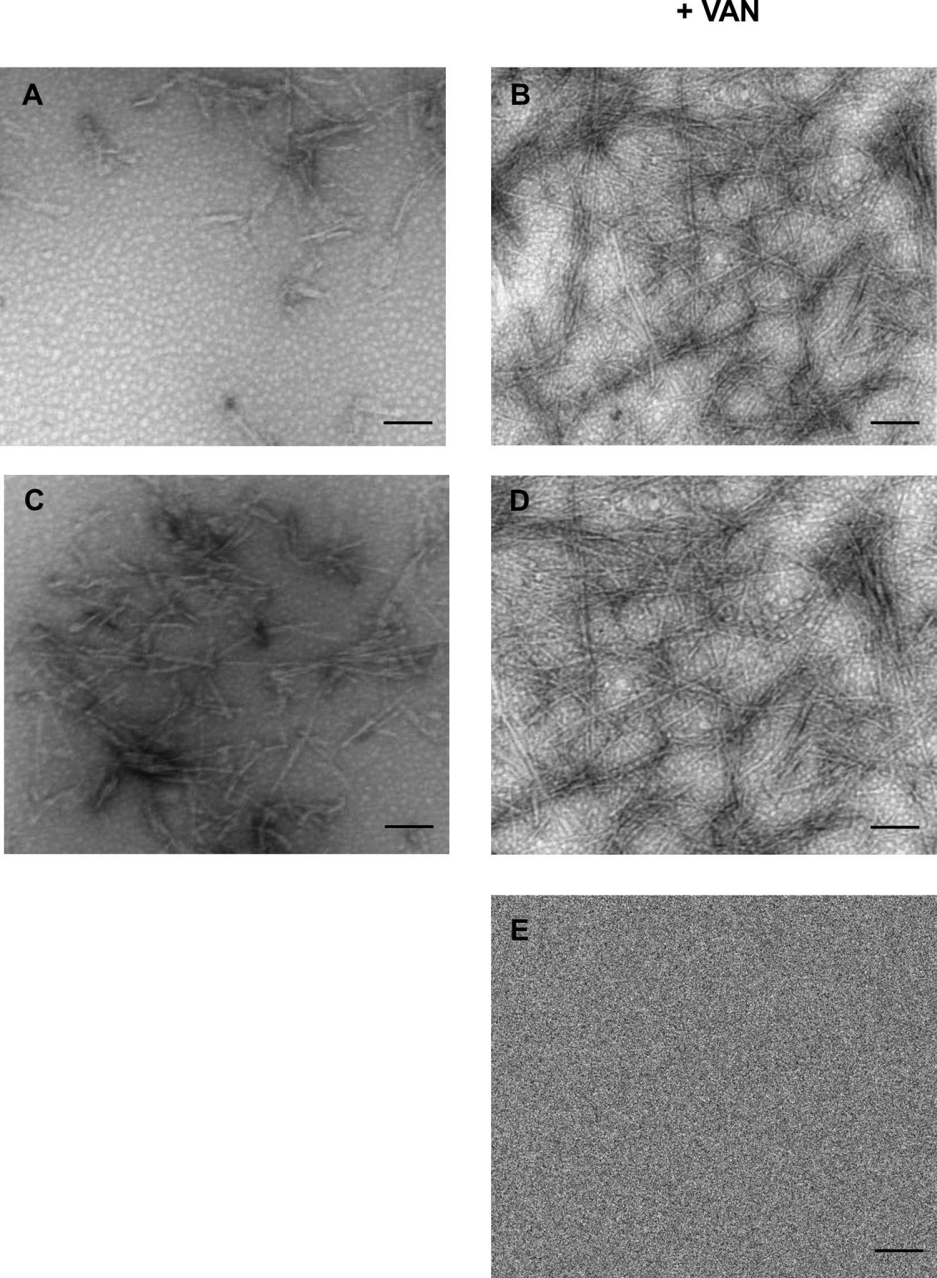
Effect of vanillin on insulin amyloid aggregation by TEM. TEM imaging of insulin samples incubated in aggregating conditions for 18 (**A**, **B**) and 24 hours (**C**, **D**) in the absence and in the presence of 100 µM vanillin. Panel **E** represents TEM imaging of vanillin. Scale bar represents 0.2 µm. Other experimental details are described in the Methods section.

In order to confirm these data, samples of insulin incubated for 18 and 24 hours in the absence and in the presence of 100 µM vanillin, were analyzed by Transmission Electron Microscopy (TEM) (Fig. 3). At 18 hours of incubation, TEM images of insulin in the absence of vanillin showed predominantly small globular species, while in the presence of vanillin fibril formation was already observed. At 24 hours, also insulin sample incubated in the absence of vanillin showed the presence of amyloid fibrils. Moreover, TEM analysis revealed a similar morphology for fibrils formed in the absence and in the presence of vanillin with a greater amount of fibrils in the sample incubated with vanillin.

### Characterization of insulin-vanillin interaction

**Figure 4:**
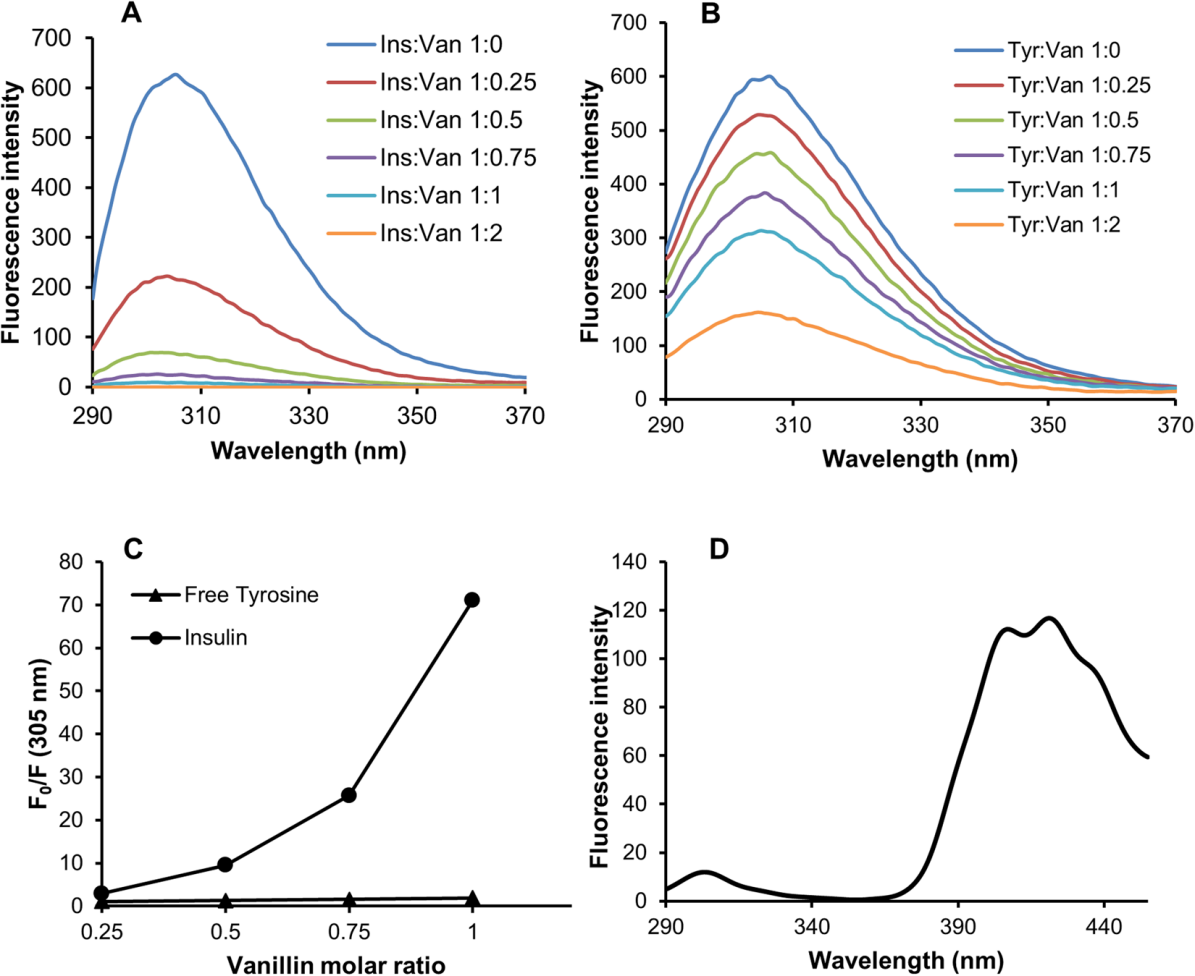
Insulin-vanillin interaction monitored by fluorescence. Tyrosine fluorescence emission was evaluated on both insulin (**A**) and free tyrosine (**B**) after addition of vanillin at different insulin:vanillin and tyrosine:vanillin molar ratio (1:0.25, 1:0.5, 1:0.75, 1:1). The dependence of F_0_/F on vanillin:insulin and vanillin:tyrosine molar ratio is shown in panel **C**. The fluorescence spectrum of insulin:vanillin (1:1) is shown in panel **D**. Working concentrations were 10 µM for insulin and 40 µM for free tyrosine. Other experimental details are described in the Methods section.

Vanillin-insulin interaction was investigated by fluorescence spectroscopy in native conditions (pH 7.0, 37 °C, without stirring). Insulin contains four tyrosyl residues as fluorescence emitters and its spectrum is characterized by the typical tyrosyl emission centered at 305 nm. The emission fluorescence spectra of insulin in the absence and in the presence of vanillin at different molar ratio are shown in Fig. 4A. The fluorescence intensity regularly decreased upon addition of increasing concentration of vanillin thus indicating that the insulin-vanillin interaction induces quenching of tyrosine emission. Interestingly, vanillin was able to strongly reduce the fluorescence intensity even at a very low molar ratio (1:0.25). The fluorescence quenching of a protein caused by small molecules may be collisional or due to the formation of a complex which has zero or small quantum yield. In order to exclude a collisional quenching by vanillin, we performed the same experiment on the monomeric tyrosyl residue (N-acetyl-L-tyrosine ethyl ester) (Fig. 4B).The F_0_/F values recorded for free tyrosine were significantly lower than those recorded for insulin at each molar ratio indicating the formation of an insulin-vanillin complex, as no collisional quenching is involved (Fig. 4C). In addition, the F_0_/F values recorded let us hypothesize a high binding affinity for the vanillin-insulin interaction in the low micromolar range. The emission spectra recorded in the presence of vanillin showed a blue-shift in the emission maximum around 301 nm indicative of a reduced polarity in the tyrosyl microenvironment. Moreover, the decrease of the fluorescence intensity at 305 nm was associated with the appearance of a new emission peak centred at 420 nm, indicative of an energy transfer between tyrosyl residues (donor) and vanillin (acceptor). Indeed, the emission spectrum of free vanillin upon excitation at 305 nm showed the appearance of an emission peak centred at 420 nm (data not shown). The formation of an insulin-vanillin complex is further supported by this evidence (Fig. 4D). A similar effect was reported for amyloid-β 1–42 upon interaction with vanillin^76^.

Analogous experiments were performed to check if also curcumin binds insulin in native conditions. The F_0_/F values recorded for insulin in the presence of increasing concentration of curcumin were comparable to those recorded for free tyrosine (Supplementary Material Fig. S2). These data indicate the occurrence of a collisional quenching thus suggesting that curcumin does not interact with insulin in native conditions.

To check if the insulin-vanillin interaction occurs through a covalent binding, we performed a mass spectrometry analysis on insulin incubated with vanillin in native conditions. No differences between insulin samples recorded in the absence and in the presence of vanillin were detected thus indicating that vanillin binds insulin through a non-covalent interaction (Supplementary Material Fig. S3). In conclusion, our data suggest that the insulin-vanillin interaction results in the formation of a non-covalent complex in which the hydrophobicity of the molecular regions surrounding tyrosyl residues is increased. This effect could involve other molecular regions affecting the overall hydrophobicity.

### Vanillin effect on insulin amyloid cytotoxicity

**Figure 5:**
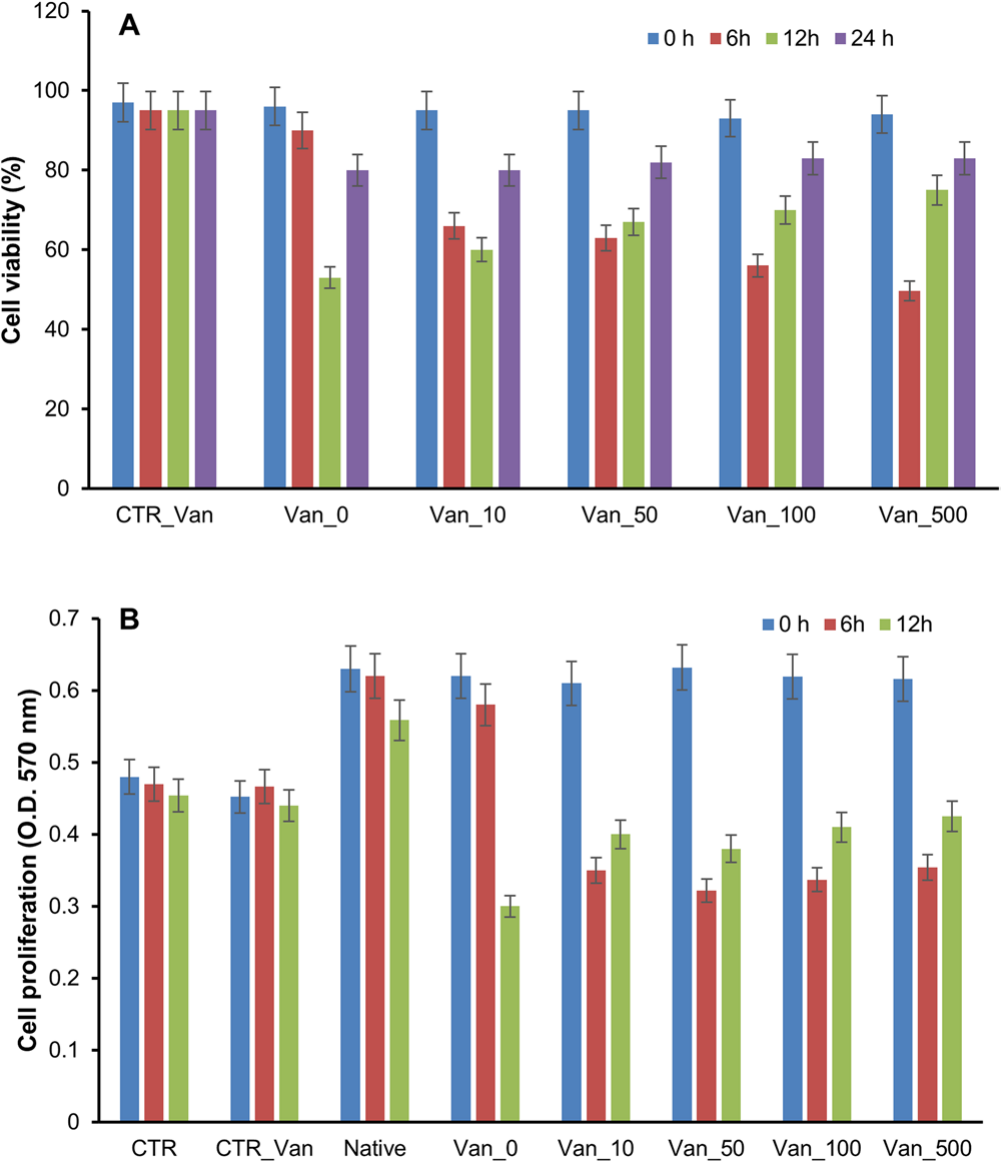
Cytotoxicity of insulin amyloid aggregates formed in the presence of vanillin. (**A**) SH-SY5Ycells were exposed for 24 hours to insulin incubated for 0, 6, 12 and 24 hours in aggregating conditions in the presence of vanillin at different concentrations (0–500 µM) and cell viability was evaluated by the MTT assay. Data are expressed as average percentage of MTT reduction ± SD relative to control cells from triplicate wells from 5 separate experiments (p < 0.01). CTR_Van represents cells exposed to vanillin at the higher working concentration. (**B**) Cell proliferation assay in cancer pancreatic cell line. Panc-1 cells were exposed for 24 hours to insulin incubated for 0, 6 and 12 hours in aggregating conditions in the presence of vanillin at different concentrations (0–500 µM) and cell proliferation was evaluated by MTT assay measuring the absorbance at 570 nm. Data are expressed as average ± SD from five independent experiments carried out in triplicate (p < 0.05). CTR: untreated cells; CTR_Van represents cells exposed to vanillin at the higher working concentration; Native: cells exposed to native insulin. Other experimental details are described in the Methods section.

Amyloid cytotoxicity is known to be associated to the early soluble oligomeric aggregates. To assess the cytotoxicity of insulin aggregates formed in the presence of vanillin, we performed both the MTT assay and cell cycle analysis. To this aim, SH-SY5Ycells were exposed for 24 hours to insulin incubated for 6, 12 and 24 hours in aggregating conditions in the presence of vanillin at different concentrations (0–10–50–100–500 µM) (Fig. 5A). As control, cell viability was assessed in the presence of insulin at the beginning of the aggregation process (time 0) and no toxicity was observed both in the absence and in the presence of vanillin at all tested concentrations. At 6 hours of incubation, while the sample incubated in the absence of vanillin did not affect cell viability, the ones in the presence of vanillin were able to induce cytotoxicity. In particular, cells exposed to insulin incubated with the higher vanillin concentration (500 µM) showed a 50% reduction of the cell viability compared to untreated cells. These data suggest that, at this time point, while insulin is mainly in a soluble not toxic conformation, samples incubated with vanillin are already in a highly toxic oligomeric state. At 12 hours of incubation also the insulin sample incubated in the absence of vanillin, induced a strong reduction of the cell viability (45%). At the same time, samples incubated with vanillin were still able to induce cell toxicity although with a minor extent respect to the 6-hour incubation. At 24 hours, all samples were not affecting cell viability indicating that the aggregates were mainly in the harmless fibril conformation. Similar indications were provided by the cell cycle analysis in which the cell toxicity is directly related to the pre-G1 content (Supplementary Material Fig. S4).

Insulin, as well as glucose, is known to promote cell proliferation in pancreatic cancer cells contributing to chemoresistance^77^. This ability was used to monitor the presence of soluble insulin for the samples aggregated in the presence and in the absence of vanillin. To this aim, cell proliferation was assessed by MTT assay on Panc-1 cells, a human pancreatic cancer line. Cells were exposed for 24 hours to insulin incubated for 0, 6 and 12 hours in aggregating conditions in the presence of vanillin at different concentrations (0–10–50–100–500 µM) (Fig. 5B). At 6 hours of incubation, as expected, native insulin was able to induce cell proliferation as indicated by the increase of the absorbance at 570 nm. The same effect was observed for insulin incubated in the absence of vanillin under aggregating conditions suggesting that the protein is still in a native-like soluble state. Differently, samples incubated in the presence of vanillin were not able to promote cell proliferation. They rather induced a further reduction of cell growth respect to untreated cells, thus suggesting that insulin is in a toxic oligomeric state. At 12 hours, cells exposed to insulin sample incubated in the absence of vanillin, showed a strong reduction of the absorbance at 570 nm indicating both loss of the proliferative activity and gain of a toxic effect likely associated to the oligomeric state. Samples incubated in the presence of vanillin were still able to reduce cell proliferation although with a minor extent respect to the previous time point. These results are consistent with the cell viability data in SHSY5Y cells and indicate that vanillin accelerates the aggregation process in insulin promoting the formation of harmless amyloid fibrils.

### Vanillin effect on insulin glycation and AGE-induced toxicity

**Figure 6:**
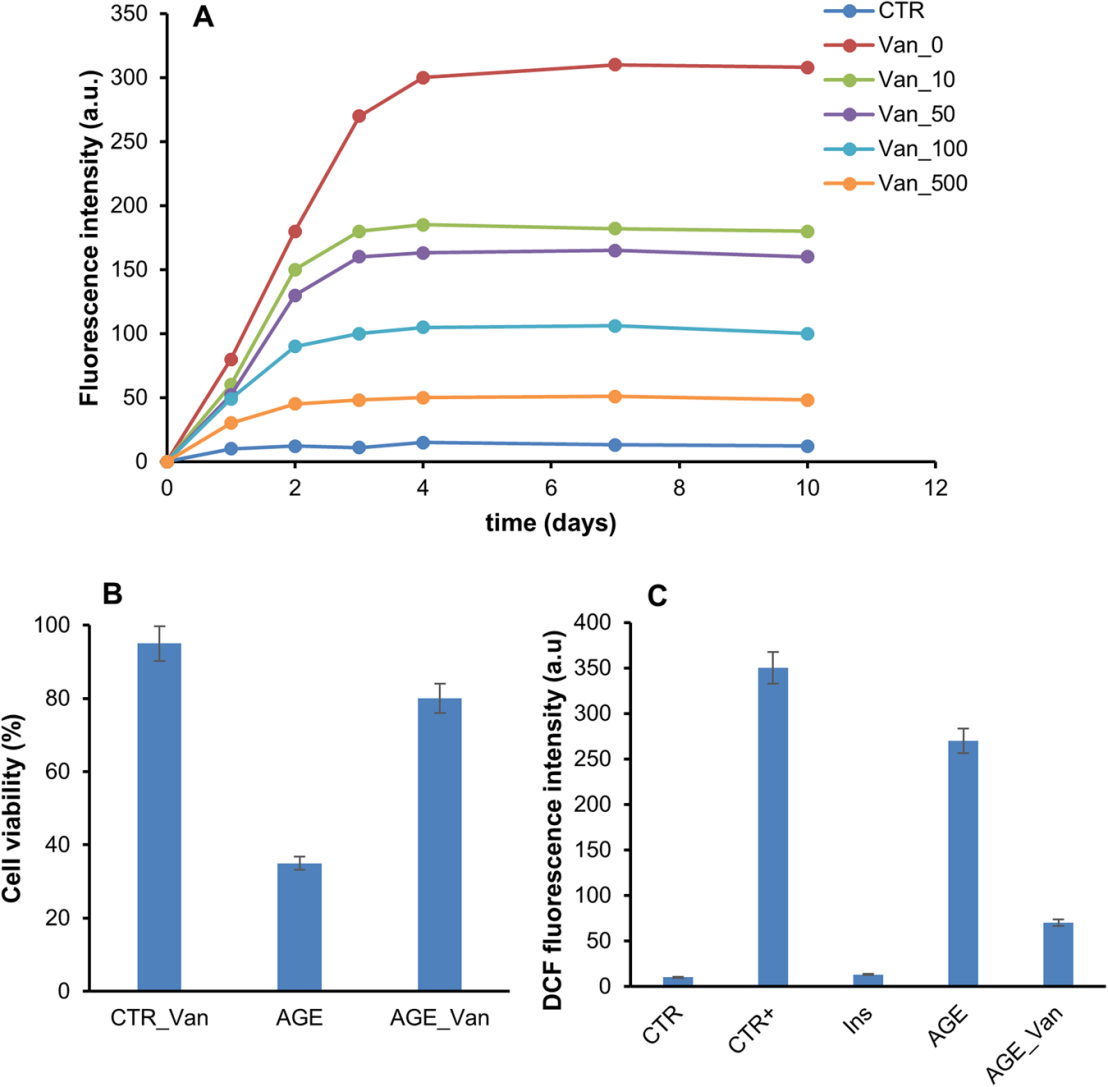
Effect of vanillin on insulin glycation kinetics and AGEs toxicity. (**A**) Insulin samples were incubated at 37 °C with 0.5 mM methylglyoxal at different concentrations of vanillin (0–500 μM) and AGE fluorescence (λ_ex_ 320 nm/λ_em_ 410 nm) was monitored at different time points. CTR: Insulin incubated in the absence of methylglyoxal. (**B**,**C**) SHSY5Y cells were exposed in the absence and in the presence of vanillin to glycated insulin and it was evaluated the cell viability after 48 h by the MTT assay (**B**) and ROS production after 72 h by the DCFH-DA assay (**C**). CTR_Van: cells treated with vanillin at the higher working concentration; CTR+: cells treated with 1.0 mM H_2_O_2_, Ins: cells treated with non-glycated insulin, AGE: cells treated with glycated insulin; AGE_Van: cells co-incubated with glycated insulin and vanillin 150 µM. For MTT experiments and DCFH-DA assay data are expressed as average percentage of MTT reduction ± SD relative to control cells from triplicate wells from 5 separate experiments (p < 0.01). Other experimental details are described in the Methods section.

Insulin is susceptible to glycation by glucose, D-ribose and other highly reactive carbonyls, such as methylglyoxal, especially in diabetic conditions and the AGE products are considered the main cause of diabetes-related vascular complications^29,31,78,79^. Methylglyoxal (MG) is the most significant glycation agent *in vivo* and its plasma levels are found increased in diabetic patients^80^. Increasing evidence suggest that curcumin possesses a protective effect against MG-induced endothelial dysfunction attenuating oxidative stress and inflammatory response^81,82^. In order to assess if also vanillin is able to interfere with AGEs formation and related toxicity, we monitored both the kinetics of AGEs formation and their ability to affect cell viability in the presence and in the absence of vanillin (Fig. 6).

Insulin glycation kinetics was monitored by fluorescence spectroscopy as AGEs are characterized by a typical fluorescence emission at 410 nm upon excitation at 320 nm. To this aim, insulin samples were incubated at 37 °C with 0.5 mM MG at different concentrations of vanillin (0, 10, 50, 100, 500 μM) and AGE fluorescence was monitored in time (Fig. 6A). In the absence of vanillin, the emission intensity at 410 nm increased markedly with incubation time and glycation reaction was completed in about 4 days. Differently, in the presence of vanillin, a drastic reduction of AGEs formation was detected at all incubation times as indicated by the decrease of fluorescence intensity. These data indicate that vanillin strongly restrains protein glycation by MG in a concentration-dependent manner. The same experiment was also performed using 0.5 M D-ribose as glycating agent but no significant effect of vanillin on protein glycation was detected (data not shown).

We have recently reported that insulin glycation by D-ribose produces AGEs adducts that strongly affect the cell viability^31^. In order to test the ability of vanillin to reduce the AGEs toxicity, we evaluated the cell viability in cells exposed to fully glycated species. In particular, we performed MTT assay on SHSY5Y cells co-incubated with ribosylated insulin and vanillin for 48 hours (Fig. 6B). As expected, glycated insulin induced a strong reduction of the cell viability (65%) while in the presence of vanillin only a 20% reduction was observed. Moreover, as AGE toxicity is generally associated to oxidative stress, the ability of vanillin to reduce ROS production was also tested^31^. To this aim, the intracellular ROS level in SHSY5Y cells co-incubated with ribosylated insulin and vanillin for 72 hours was measured by DCFH-DA fluorescence assay (Fig. 6C). Interestingly, while ribosylated insulin promotes ROS production as indicated by the increase in the DCF fluorescence, in the sample co-incubated with vanillin a strong reduction of ROS levels was observed. These data suggest that vanillin exerts a protective effect in AGE-induced cytotoxicity likely affecting death pathways mediated by intracellular ROS production.

## Discussion

Curcumin is known for many properties, including anti-inflammatory, antioxidant, and anticancer activity that make it an attractive potential drug^43–46^. Also, curcumin has been shown to affect amyloid aggregation and AGE formation in several model proteins^34–42^. Nevertheless, its potential therapeutic use seems to be limited because of its very low systemic bioavailability after oral administration owing to its low water solubility and its chemical instability under physiological or alkaline conditions^44,47–49^. Indeed, the 90% of curcumin undergoes rapid hydrolysis in aqueous solution leading to several degradation products, identified as trans-6-(4′-hydroxy-3′-methoxyphenyl)-2,4-dioxo-5-hexenal, ferulic aldehyde, ferulic acid, feruloyl methane, and vanillin^49^. Despite the high instability, curcumin is known to be highly beneficial for human health. For this reason, recently much attention has been paid to the biological properties of the degradation products of the curcumin as it has been suggested that they may contribute to its pharmacological effects^51,54–57^. Between them, vanillin seems to be very promising as it represents one of the most stable degradation product of curcumin in physiological conditions. Recently, it has been reported that vanillin is able to restrain non-enzymatic glycation and AGE-induced amyloid aggregation in albumin^62^. However, little information is available on the effect of vanillin in amyloid aggregation and AGE formation. In order to add knowledge in this field and elucidate the molecular basis for a potential therapeutic use of vanillin, we have investigated the effect of vanillin in both insulin amyloid aggregation and AGE formation.

Insulin is a protein mainly organized in helical structure and its amyloid aggregation process occurs via a nucleation dependent mechanism in which the early aggregates, still retaining a helical structure, eventually lead to the formation of amyloid fibrils showing the typical cross-β-structure^69,70^. Our results suggest that vanillin strongly affects the amyloid aggregation process in human insulin. Indeed, CD analysis, Congo red assay and TEM imaging indicate that vanillin favors the α to β transition underlying the amyloid assembly thus accelerating fibril formation. The oligomeric aggregates formed in the early stage of the fibrillation process are considered the main responsible for amyloid toxicity, whereas amyloid fibrils are essentially harmless^73^. However, molecules that both inhibit protein fibrillization and stabilize the aggregates in a non-toxic state can be considered in potential therapeutic application. In this respect, vanillin could strongly mediate amyloid toxicity accelerating the kinetics of the harmless fibril formation. Similarly, the presence of sub-stoichiometric amount of curcumin was able to accelerate amyloid fibril formation in human insulin in the same aggregation conditions (pH 7.0, 37 °C).

Our results suggest, for insulin, a different effect to that reported for other amyloidogenic proteins. Indeed, curcumin has been shown to have an anti-amyloidogenic effect for Aβ-peptide, IAPP, α-synuclein and lysozyme^34–41^. It should be taken into account that the inhibition of amyloid aggregation by curcumin seems to be mediated by specific interactions with protein surface and aromatic residues thus depending on the conformational properties^41,83^. Also for bovine insulin in aggregating conditions at pH 2.5, curcumin was shown to reduce amyloid fibril formation^42^. However, the amyloid aggregation process of insulin is known to be highly susceptible to environmental conditions such as pH, temperature, protein concentration and ionic strength able to promote different aggregation pathways^66,67,84^. Specifically, insulin fibrillation under neutral pH conditions occurs via a different pathway from that under acidic conditions^85^. At pH 2.5 the amyloid aggregation originates from a conformational change leading the monomeric state into a partially folded intermediate responsible for the formation of the early aggregating nuclei. Differently, at neutral pH, the most populated association state is the dimeric form that evolves in the early aggregating nuclei likely via different intermediate states^85^. In this respect, we could hypothesize that, at pH 2.5, curcumin stabilizes the monomeric form thus hindering the molecular transitions underlying the formation of the early aggregating nuclei and inhibiting the fibril formation. At pH 7.0 curcumin could not efficiently interact with the dimeric form but rather with the intermediates states on the aggregation pathway thus favouring amyloid aggregation. Indeed, the intrinsic fluorescence data indicated that curcumin does not interact with insulin in native conditions. Compared to curcumin, vanillin is a much smaller molecule and, for this reason, it could easily interact with insulin polar or charged residues likely through the OH- group thus exposing its aromatic ring and increasing the hydrophobicity in the binding region. Intrinsic fluorescence experiments showed the formation of an insulin-vanillin complex. Interestingly, upon binding to vanillin, the fluorescence emission maximum was blue-shifted suggesting a reduced polarity in the tyrosyl microenvironment likely due to an increased hydrophobicity of the molecular regions surrounding tyrosyl residues. The insulin-vanillin complex is not stabilized by covalent interactions as revealed by mass spectrometry analysis thus suggesting that hydrogen bonds as well as van der Waals forces are involved. The increased hydrophobicity could be responsible for the faster fibril formation observed in the presence of vanillin as it likely promotes protein association in aggregating conditions favoring the formation of the early aggregating nuclei.

However, the similar effects observed for curcumin and vanillin in insulin amyloid formation support the hypothesis that vanillin could mediate the curcumin biological properties. Moreover, our results indicate that the effects of curcumin, as well as vanillin, cannot be generalized as depending by molecular interactions between these molecules and specific residues exposed on the protein structure. For this reason, different effects could be observed in relation to different polypeptide chain properties.

Similarly, our results indicate that vanillin could mediate the anti-AGEs activity of curcumin. Indeed, the AGE formation by MG was strongly reduced in the presence of vanillin. Recently, it has been reported that curcumin inhibits the glycation reaction by specifically trapping the MG^81,82^. As vanillin affects insulin glycation in the presence of MG and not D-ribose, we can hypothesize that also vanillin might directly trap MG further suggesting that curcumin degradation products could strongly contribute to the anti-AGE activity observed for curcumin.

In insulin glycation, MG is known to react with a single site, i.e., Arg22 of insulin B chain^29^. In this respect, the anti-AGE activity observed for vanillin could also be ascribed to a direct non-covalent interaction between vanillin and the positively charged Arg22 side chain that would hinder the glycation reaction.

Moreover, our results indicate that vanillin exerts a protective effect in AGE-induced cytotoxicity. Recently, we reported that ribose-glycated insulin strongly affects the cell viability, promoting death pathways involving oxidative stress, apoptosis and inflammatory response activation^31^. Vanillin is known for its antioxidant activity and possess a significant brain-protective effect against oxidative damage^86,87^. Our data show that vanillin reduces the AGEs induced ROS production when co-incubated with fully ribosylated insulin. In this respect, we can suggest that vanillin exerts its protective action affecting death pathways mediated by intracellular ROS production.

Finally, the overall data contribute to validate the hypothesis that curcumin degradation products, like vanillin, are the main bioactive molecules in achieving the biological activities of curcumin. Our data are consistent with previous observations indicating that curcumin and its degradation products possess similar anti-cancer, anti-inflammation and anti-microbial activity.

Our findings open new avenues for developing therapeutic applications for vanillin, especially in consideration of its good safety profile being a natural product, and the ability to cross the blood brain barrier.

## Methods

### Materials

Human insulin, curcumin, vanillin, Thioflavin T, N-acetyl-L-tyrosine-ethyl ester, methylglyoxal, D-ribose, 3-(4,5-dimethylthiazol-2-yl)-2,5-diphenyl-tetrazolium bromide (MTT) (Sigma-Aldrich Co., St. Louis, MO). Uranil acetate replacement stain (Electron Microscopy Sciences, Hatfield, PA). All other chemicals were of analytical grade. Methylglyoxal was further purified by distillation under low pressure and its concentration was determined spectrophotometrically using ε_284_ = 12.3 M^−1^ cm^−1^ ^88^.

### Insulin preparation and amyloid aggregation

Human insulin was dissolved in ultra-pure milliQ water to a final concentration of 4 mg/ml at pH 2.0 in order to obtain monomeric insulin; protein concentration was determined by absorbance (ε_275_ = 4560 M^−1^cm^−1^). Finally, insulin was neutralized to pH 7.0 and kept in phosphate buffer 50 mM, pH 7.0. Curcumin was dissolved in 100% DMSO at 2 mM concentration; vanillin was dissolved in ethanol at 100 mM concentration. For aggregation studies, protein samples were diluted at a final concentration of 0.5 mg/mL and incubated at 37 °C under vigorous stirring with teflon balls, 1/8″ diameter (Polysciences, Inc.) in the absence and in the presence of 10 and 100 µM curcumin or 10, 50, 100, and 500 µM vanillin. Aliquots of protein were collected in sterile conditions and immediately analyzed.

### Insulin glycation

Glycated insulin was prepared mixing human insulin at a final concentration of 0.5 mg/mL and 0.5 mM methylglyoxal or 0.5 M D-ribose in 50 mM NaH_2_PO_4_ buffer, pH 7.0, passed through a 0.22 μm filter and incubated at 37 °C in sterile conditions in the absence and in the presence of 10, 50, 100, and 500 µM vanillin. Human insulin in buffer without glycating agent was used as protein control.

### Circular Dichroism measurements

CD spectra were recorded at 25 °C on a JascoJ-715 spectropolarimeter using thermostated quartz cells of 0.1 cm. Spectral acquisition was taken at 0.2 nm intervals with a 4-sec integration time and a bandwidth of 1.0 nm. An average of five scans was obtained for all spectra. Photomultiplier absorbance did not exceed 600 V in the spectral region analyzed. All measurements were performed under nitrogen flow and spectra were recorded after diluting of the samples at a final protein concentration of 0.3 mg/mL. Data were corrected for buffer contributions using the software provided by the manufacturer (System Software version 1.00) and transformed in mean residue ellipticity.

### Fluorescence measurements

Fluorescence measurements were performed on a Perkin Elmer Life Sciences LS 55 spectrofluorimeter. Thioflavin T (ThT) fluorescence (λ_ex_ 450 nm/λ_em_ 482 nm) was monitored at different time intervals after addition of ThT to protein samples. Working concentrations were 8 µM for protein samples and 25 µM for ThT. As control, the emission intensity of the samples in the presence and in the absence of curcumin was measured before the addition of ThT. Only a very low intensity was detected for all samples and it was subtracted to the emission intensity recorded after the addition of ThT. Tyrosine fluorescence emission (λ_ex_ 275 nm/λ_em_ 305 nm) was evaluated on both insulin and free tyrosine after addition of vanillin at different insulin:vanillin molar ratio (1:0.25, 1:0.5, 1:0.75, 1:1). Tyrosil fluorescence quenching was monitored by extimation of the F_0_/F ratio considering the fluorescence intensity at 305 nm of the sample before (F_0_) and after (F) the addition of vanillin. Working concentrations were 10 µM for insulin and 40 µM for free tyrosine. To assess the intrinsic fluorescence of AGEs (λ_ex_ 320 nm/λ_em_ 410 nm), glycated insulin at a final concentration of 8 μM was monitored at different incubation times with the glycating agent in the absence and in the presence of vanillin. The fluorescence intensity was corrected by subtracting the emission intensity of D-ribose/methylglyoxal solutions at different incubation times.

### Congo Red Binding Assay

For this assay 8 µM insulin and 5 µM Congo Red were incubated at room temperature for 30 minutes before recording the absorption spectra. Congo Red stock solution (5.0 mM) was prepared in phosphate buffer 20 mM, pH 7.4. Spectra were acquired on Jasco V-550 spectrophotometer in the 400–600 nm region.

### Transmission electronic microscopy (TEM)

Aliquots of protein samples (3 µL) were placed on the copper grid and allowed to dry. The remaining liquid is removed using filter paper. After 5–6 minutes uranil acetate replacement stain 1X (3 µL) was loaded on the grid and air dried. Images were acquired using a Libra 120 (Zeiss) Transmission Electron Microscope equipped with Wide-angle Dual Speed CCD-Camera sharp:eye 2 K (4Mpx.).

### Cell cultures and treatments

SH-SY5Y human neuroblastoma cells (ATCC# CRL-2266) were cultured in Eagle’s Minimum Essential Medium (EMEM) supplemented with 10% fetal bovine serum, 3.0 mM glutamine, 50 units/mL penicillin and 50 mg/mL streptomycin in a 5.0% CO_2_ humidified environment at 37 °C. PANC-1 cells (ATCC# CRL-1469) were cultured in Dulbecco’s modified eagle’s medium (DMEM) – high glucose supplemented with 10% (v/v) heat inactivated fetal bovine serum, 50 units/mL penicillin and 50 mg/mL streptomycin at 37 °C in a humidified atmosphere with 5% CO_2_. The cells were plated at a density of 100,000 cells/well on 24-well plates in 1 ml of medium. After 24 h, cells were exposed to 30 µM protein samples. Cells in culture medium without protein and in the presence of curcumin and vanillin at the tested concentrations served as control. In the AGE experiments, before cells exposure, insulin glycated in the presence of 0.5 M D-ribose for 8 days was subjected to dialysis in sterile conditions to remove the free glycating agent^89^.

### MTT assay

Cell viability was assessed as the inhibition of the ability of cells to reduce the metabolic dye 3-[4,5-dimethylthiazol-2-yl]-2,5-diphenyltetrazolium bromide (MTT) to a blue formazan product^90^. After indicated times of incubation with protein samples, cells were rinsed with phosphate buffer solution (PBS). A stock solution of MTT (5 mg/mL in PBS) was diluted ten times in cell medium and incubated with cells for 3 hours at 37 °C. After removing the medium, cells were treated with isopropylalcohol, 0.1 M HCl for 20 min. Levels of reduced MTT were assayed by measuring the difference in absorbance between 570 and 690 nm. Data are expressed, as average percentage reduction of MTT with respect to the control ± S.D. Data are an average from five independent experiments carried out in triplicate. In cell proliferation assay the MTT absorbance was evaluated at 570 nm.

### Detection of intracellular ROS

Intracellular ROS were detected by means of an oxidation-sensitive fluorescent probe 2′,7′-dichlorofluorescin diacetate (DCFH-DA). Cells were grown in a12-well plates, pre-incubated with DCFH-DA for 30 min and then incubated with protein samples for 72 hours. Control experiments were performed using untreated cells and cells exposed to a 0.001 M H_2_O_2_. After incubation, cells were washed twice with PBS buffer and then lysed with Tris-HCl 0.5 M, pH 7.6, 1% SDS. The non-fluorescent DCFH-DA is converted, by oxidation, to the fluorescent molecule 2′,7′-dichlorofluorescein (DCF). DCF fluorescence intensity was quantified on a Perkin Elmer Life Sciences LS 55 spectrofluorimeter using an excitation wavelength of 488 nm and an emission wavelength of 530 nm. Data are expressed as average ± S.D. from five independent experiments carried out in triplicate.

### Statistical analysis

For statistical analysis, we used a two-tailed Student’s t test with unequal variance at a significance level of 5% unless otherwise indicated.

### Data Availability

The datasets generated and/or analysed during the current study are available from the corresponding author on reasonable request.

## Electronic supplementary material

Supplementary material
