# Supplementary material for: Vanillin Affects Amyloid Aggregation and Non-Enzymatic Glycation in Human Insulin
Source: Sci Rep. 2017 Nov 8;7:15086. doi: 10.1038/s41598-017-15503-5 (PMC5678370; doi:10.1038/s41598-017-15503-5)
Supplement: Supplementary file 1 — Supplementary material [file 41598_2017_15503_MOESM1_ESM.docx]

**VANILLIN AFFECTS AMYLOID AGGREGATION AND NON-ENZYMATIC GLYCATION IN HUMAN INSULIN**

**Clara I****annuzzi^1^, Margherita Borriello^1^, Gaetano Irace^1^, Marcella Cammarota^2^, Antimo Di Maro^3^, and Ivana Sirangelo^1,*^**

^1^Department of Biochemistry, Biophysics and General Pathology, Università degli Studi della Campania “Luigi Vanvitelli”, Naples, 80138, Italy

^2^ Department of Experimental Medicine, Università degli Studi della Campania “Luigi Vanvitelli”, Naples,80138, Italy

^3^Department of Environmental, Biological and Pharmaceutical Sciences and Technologies, Università degli Studi della Campania “Luigi Vanvitelli”, Caserta, 81100, Italy

^*^Corresponding author: [ivana.sirangelo@unicampania.it](mailto:ivana.sirangelo@unicampania.it)

**Supplementary Material 1**

**
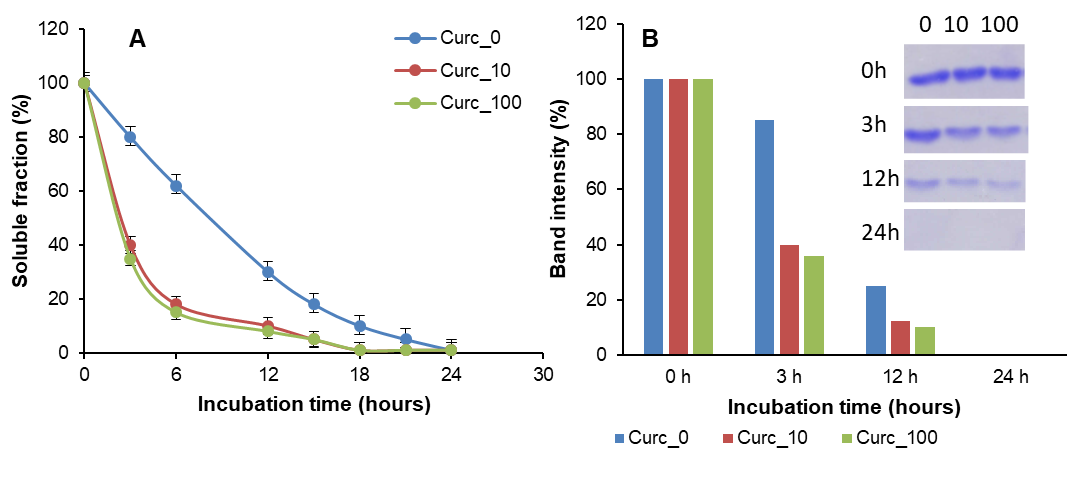
**

**Figure S1**: Aggregation kinetics of insulin in the presence of curcumin monitored by absorbance spectroscopy and SDS-PAGE analysis. The soluble fraction was monitored during the aggregation process at different incubation times for insulin in the absence (Curc_0) and in the presence of 10 µM (Cur_10) and 100 µM (Cur_100) curcumin. The protein stock solution (0.5 mg/mL) was centrifuged at different times for 30 minutes at 13000 x g at 4 °C and supernatant was analyzed by UV-absorption (A) and SDS-PAGE (B) analysis. The protein concentration was estimated by absorbance at 275 nm (ε_275_= 4560 M^-1^cm^-1^) and the soluble fraction is reported in percentage respect to the protein in native conditions. The SDS-PAGE densitometric analysis was performed using Image Quant TL (IQTL) software (GE healthcare). The band intensity is reported in percentage respect to the protein in the soluble fraction at time 0.

**Supplementary Material 2**


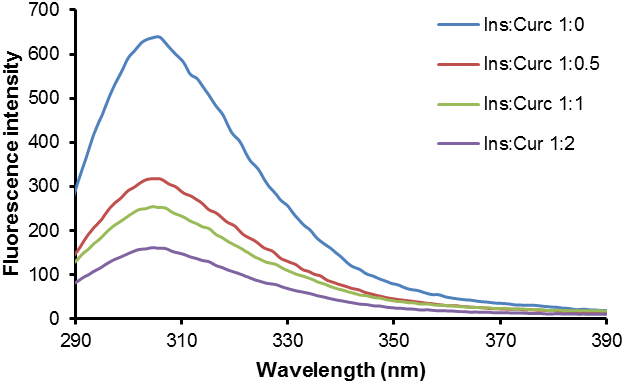


**A**

**B**


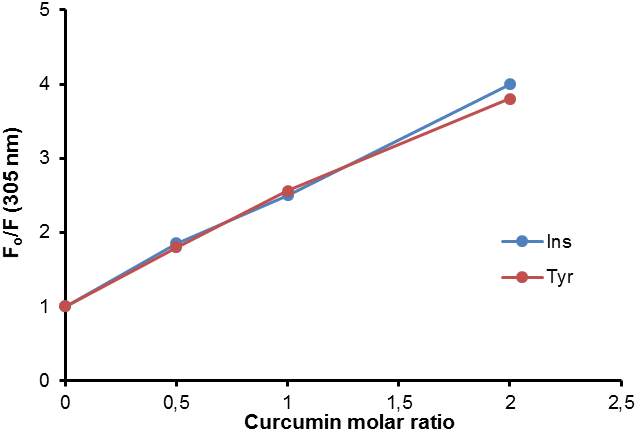


**Figure S2:** (A) Tyrosine fluorescence emission of human insulin after addition of curcumin at different insulin:curcumin molar ratio (1:0, 1:0.5, 1:1, 1:2). (B) Comparison between the F0/F values obtained for insulin (blue line) and free tyrosine (red line) at different curcumin molar ratio. Working concentrations were 10 µM for insulin and 40 µM for free tyrosine.

**Supplementary Material 3**


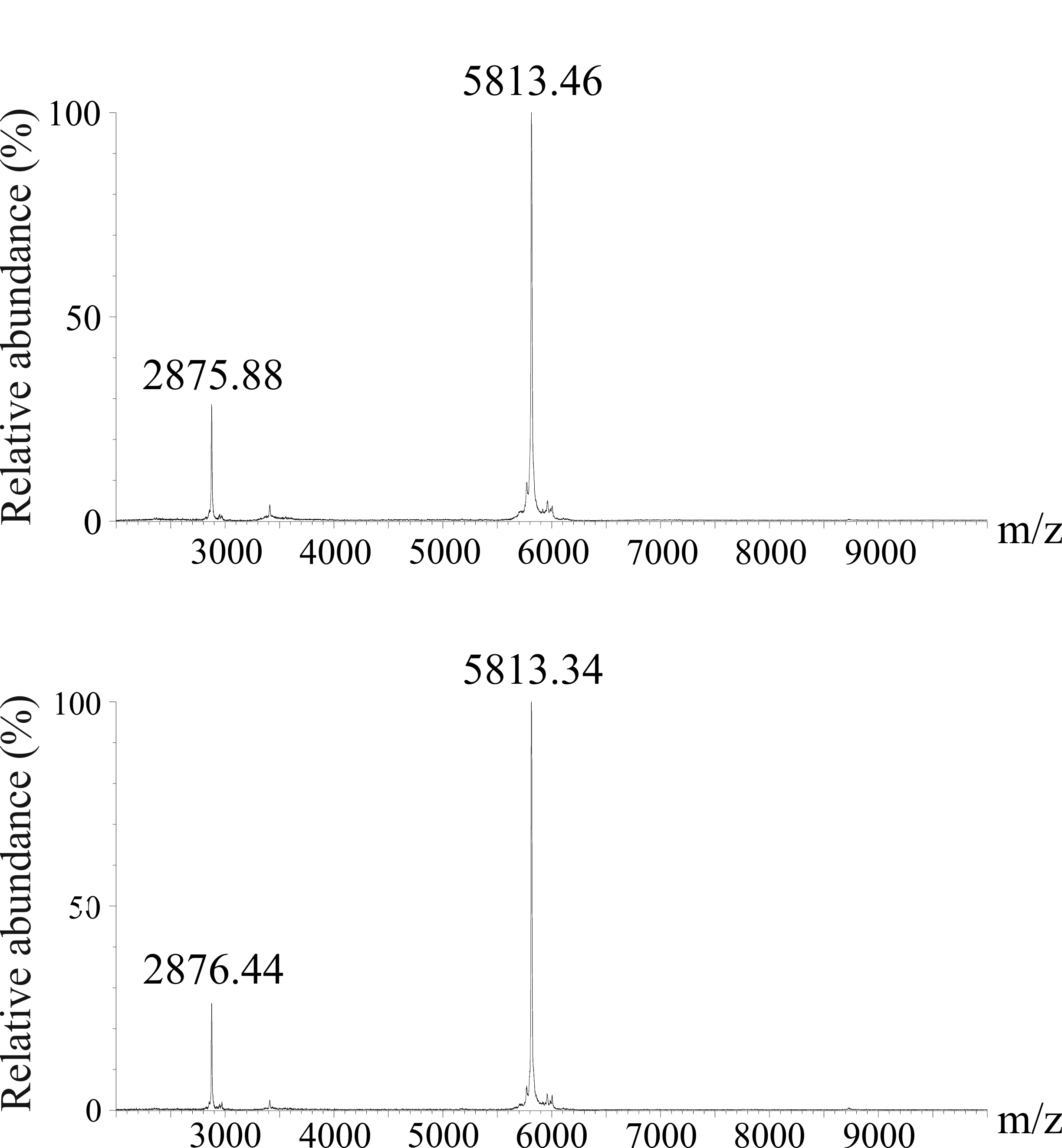


**A**

**B**

**Figure S3:** Insulin-vanillin interaction monitored by mass spectrometry. MALDI-TOF mass spectrum of insulin in the presence (panel A) and in the absence (panel B) of vanillin in 1:1 molar ratio. The peak at about 2876 Da corresponds to the doubly charged ion [M+2H]2+.

**Methods**

**Mass Spectrometry analysis.** Mass Spectrometry analysis was performed on a MALDI-TOF micro MX instrument (Waters Co., Manchester, UK) equipped with a pulsed nitrogen laser (λ = 337 nm). The instrument source voltage was set to 12 kV. The pulse voltage was optimized at 1999 V, and the detector voltage was set to 5200 V. Prior to acquisition of spectra, 1 μL of insulin solution was mixed with 1 μL of saturated α-cyano-4-hydroxycinnamic acid matrix solution [10 mg/mL in acetonitrile:0.1% TFA (1:1; v/v)]. A droplet (1 μL) of the resulting mixture was placed on the mass spectrometer’s sample target and dried at room temperature. After complete evaporation of the liquid, the sample was loaded into the mass spectrometer and analysed in positive acquisition linear mode. External calibration was with a mixture of standard proteins (10 pmol/1 mL each of insulin, cytochrome c, horse Mb and trypsinogen; Sigma). Mass accuracy near the nominal value (300 ppm) was achieved for each standard. All spectra were processed and analysed using MassLynx version 4.1 software (Waters, Milford, MA).

**Supplementary Material 4**


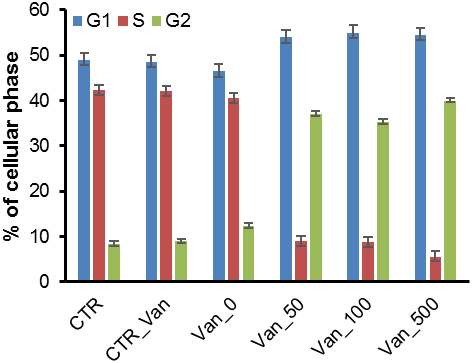


**A**


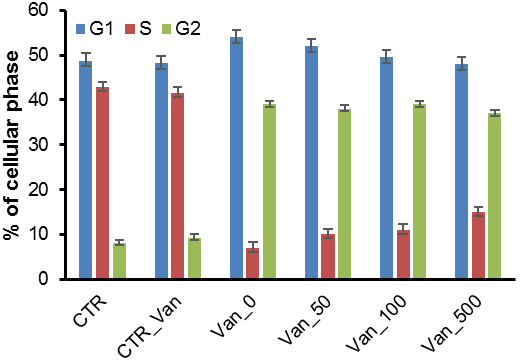


**B**


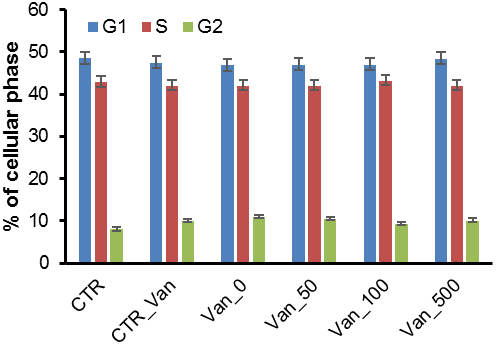


**C**


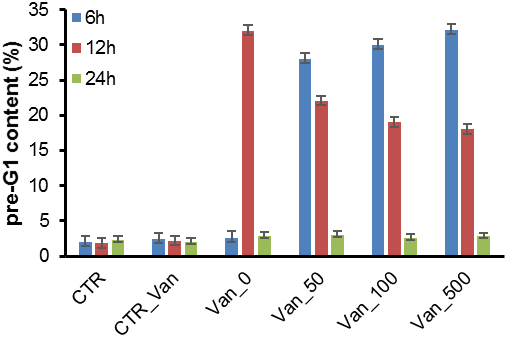


**D**

**Figure S4.** Cell cycle analysis of SH-SY5Ycells exposed for 24 hours to insulin incubated for 6 (panel A), 12 (panel B) and 24 hours (panel C) in aggregating conditions in the presence of vanillin at different concentrations (0-500 µM). Analysis of the pre-G1 content is shown in panel D. CTR: untreated cells; CTR_Van represents cells exposed to vanillin at the higher working concentration. Data are expressed as average ± SD from five independent experiments carried out in triplicate (p<0.01).

**Methods**

**Cell-cycle analysis.** After 24 hours of incubation with protein samples, 2.5x10^5^ cells were collected and resuspended in 500μL of hypotonic buffer (0.1% Triton X-100, 0.1% sodium citrate, 50 μg /mL PI, RNAse A). Cells were incubated in the dark for 30 minutes and samples were acquired on a FACS-Calibur flow cytometer using the Cell Quest software (Becton Dickinson) and ModFitLT version 3 software (Verity).
